# Survey data on employees’ development and employees’ satisfaction in oil and gas firms in Nigeria

**DOI:** 10.1016/j.dib.2018.06.066

**Published:** 2018-06-28

**Authors:** Bolanle D. Motilewa, Christiana O. Bisi-Adeniyi, Oluwaseyi A. Fambegbe, Adeola I. Oyeyemi, Rowland E.K. Worlu, Chinonye L. Moses

**Affiliations:** a Business Management Department, Covenant University, Ota, Nigeria; b Psychology Department, Covenant University, Ota, Nigeria; c Political Science and International Relations Department, Covenant University, Ota, Nigeria

**Keywords:** Employees’ development, Employees’ satisfaction, Corporate social responsibility, Organisational performance, Oil and gas firms, Nigeria

## Abstract

Employees’ development involves ensuring that employees are compensated fairly, are not exposed to dangerous or environmentally unhealthy working environment and are treated ethically in the workplace, especially in a technology intense industry as that of the oil and gas sector. Thus, this article presents data on the effect of employees’ development on employees’ satisfaction. The study employed a descriptive quantitative research design engaging survey method. The study population consists of 1748 employees from four top oil and gas firms quoted in the Nigerian stock exchange. A sample size of 350 employees was selected. Data was analysed using statistical Package for Social Sciences (SPSS). Regression analysis was employed as the statistical tool of analysis. The field data set is made widely accessible in this article.

**Specification Table**

**Table:** 

**Subject area**	Business and Management
**More Specific Subject Area**	Human Resources Management, Industrial Psychology, Corporate Social Responsibility,
**Type of Data**	Tables
**How Data was Acquired**	Survey
**Data Format**	Raw, Filtered and Descriptive
**Experimental Factors**	A simple random sampling technique was used to gather the data
**Experimental features**	The gathered data were based on randomly selected respondents among employees in oil and gas firms operating in Lagos.
**Data source location**	Lagos, Nigeria
**Data Accessibility**	Data is provided with this article

**Value of Data**

• The data provided gives an insight on the firms’ involvement in employees’ development within the confines of corporate social responsibility in oil and gas firms in Nigeria. Further studies can review this stance in other industries.
• The original data file may be used for studying similar issues at individual employee level.
• The provided data also shows statistics on CSR in the developing country׳s perspective. Considering the limited available data on CSR that goes beyond philanthropy from the developing country׳s perspective, future studies might consider expanding their investigation into other aspects of CSR beyond philanthropy.
• Considering the limited available data on employees’ perception of the firm׳s commitment to employee related corporate social responsibility practices, this data set opens up avenue for future studies focused on implicit CSR.

## 1 Data

A total of three hundred and fifty copies of questionnaire were administered to respondents from the top four listed oil and gas firms in Nigeria׳s stock exchange. Table 1 below shows that 22.9% of the population of this study were from Firm 1, 27.3% from Firm 2, 27.8% from Firm 3 and 22% from Firm 4. This clearly shows that each firm for the study was well represented. The demographic characteristics of the respondents are also highlighted in Table 2 below.

**Table 1:** Sample frame for distribution of questionnaire. Source: Researcher׳s Field Survey, 2017.

**Name of firm**	**Number of employees**	**Percentage of total (%)**		**Questionnaire Distributed**
Firm 1	401	401/1748*100	22.9	80
Firm 2	477	477/1748*100	27.3	96
Firm 3	485	485/1748*100	27.8	97
Firm 4	385	385/1748*100	22.0	77
**Total**	**1748**		**100**	**350**

**Table 2:** Demographic characteristics of respondents. Source: Researcher׳s Field Survey, 2017.

**Demographic Characteristics**	**Items**	**Oil and Gas Firms**				**Total**
		**Firm 1**	**Firm 2**	**Firm 3**	**Firm 4**	
		**(%)**	**(%)**	**(%)**	**(%)**	
**Gender**	Male	38	40	59	57	194
		(49.4)	(44.4)	(62.8)	(76.0)	(57.7)
	Female	39	50	35	18	142
		(50.6)	(55.6)	(37.2)	(24.0)	(42.3)
**Total**		**77**	**90**	**94**	**75**	**336**
**Age**	Under 25 yrs	–	9	–	11	20
			(10.0)		(14.7)	(5.9)
	25–35 yrs	36	52	23	33	144
		(46.7)	(57.8)	(24.5)	(44.0)	(42.9)
	36–45 yrs	21	17	67	31	136
		(27.3)	(18.9)	(71.3)	(41.3)	(40.5)
	46 yrs +	20	12	4	–	36
		(26.0)	(13.3)	(4.3)		(10.7)
**Total**		**77**	**90**	**94**	**75**	**336**
**Length of Service**	Less than 5 yrs	57	51	70	22	200
		(74.0)	(56.7)	(74.5)	(29.3)	(59.5)
	5–10 years	20	21	20	26	87
		(26.0)	(23.3)	(21.3)	(34.7)	(25.9)
	11–15 years	–	4	4	25	33
			(4.4)	(4.3)	(33.3)	(9.8)
	16 yrs and above	–	14	–	2	16
			(15.6)		(2.7)	(4.8)
**Total**		**77**	**90**	**94**	**75**	**336**
**Status or Position**	Director	–	–	–	–	–
	Senior Manager	20	9	44	2	75
		(26.0)	(10.0)	(46.8)	(2.7)	(22.3)
	Analyst	–	28	17	23	68
			(31.1)	(18.1)	(30.7)	(20.2)
	Supervisor	57	8	2	41	108
		(74.0)	(8.9)	(2.1)	(54.7)	(32.3)
	Others	–	45	31	9	85
			(50.0)	(33.0)	(12.0)	(25.3)
**Total**		**77**	**90**	**94**	**75**	**336**
**Educational Status**	OND/NCE	–	13	–	7	20
			(14.4)		(9.3)	(5.9)
	HND/BSc	38	36	60	27	161
		(49.4)	(40.0)	(63.8)	(36.0)	(47.9)
	MSc/MBA/MEd	39	26	31	37	133
		(50.6)	(28.9)	(33.0)	(49.3)	(39.6)
	Others	–	15	3	4	22
			(16.7)	(3.2)	(5.3)	(6.6)
**Total**		**77**	**90**	**94**	**75**	**336**

### 1.1 Statement of test statistics

Given that the correlation co-efficient measures the degree to which two things vary together, this model correlated two variables: employees’ development and employees’ satisfaction.

Table 3 shows the descriptive statistics of employees’ development and employees’ satisfaction for each oil and gas firm. All the sampled firms agreed with all the constructs in this variable. Most of the respondents acknowledged positively to the contribution of employees’ development and employees’ satisfaction. Nonetheless all the respondents (Firm 4 = 4.671, Firm 1 = 4.338, Firm 3 = 4.200 and Firm 2 = 4.196) admitted favourably with mean scores above 4.000 to the statement. This indicates that management of the sampled firms should continue to formulate and implement policies targeted towards improving their employees’ development.

**Table 3:** Descriptive statistics of variables for each oil and gas firm. Source: Researcher׳s Field Survey, 2017.

**Descriptive Statistics**								
	**Firm 1**		**Firm 2**		**Firm 3**		**Firm 4**	
	**Mean**	**SD**	**Mean**	**SD**	**Mean**	**SD**	**Mean**	**SD**
**Employees’ Development**	4.338	.266	4.196	.501	4.200	.349	4.671	.341
**Employees’ Satisfaction**	4.312	.273	4.031	.509	4.178	.409	4.580	.397
	**Freq = 77**		**Freq = 90**		**Freq = 94**		**Freq = 75**	

^a^ Predictors: (Constant), Employees’ development: *training, health and safety, Employees’ inclusiveness, policies against discrimination, work-life balance*.

^b^ Dependent variable: Employees’ Satisfaction.

The above Table 4 shows the statistical significance of the two variables for each oil and gas firm: employees’ development and employees’ satisfaction using the multiple regression. The statistics presented in the table above under *R* square is called the coefficient of determination and referred to as *R*^2^. The *R* Square tells how much of the variance in the dependent variable (employee satisfaction) is explained by the independent variable (employees’ development). The *F* statistic tests the overall significance of the model. In this case, the value for each firm (Firm 1 = .189, Firm 2 = .553. Firm 3 = .576 and Firm 4 = .570) is expressed as a percentage, this means that the independent variable (employees’ development) explains Firm 1 (18.9%):, Firm 2 (55.3%), Firm 3 (57.6%) and Firm 4 (57%) of the variance in employees’ satisfaction. Table 5.

**Table 4:** Model characteristics for each firm. Source: Researcher׳s Field Survey, 2017.

**Firm 1**			**Firm 2**			**Firm 3**			**Firm 4**		
***r***	***r***^**2**^	**Sig.**	***r***	***r***^**2**^	**Sig.**	***r***	***r***^**2**^	**Sig.**	***r***	***r***^**2**^	**Sig.**
.434^a^	.189	.000^b^	.743^a^	.553	.000^b^	.759^a^	.576	.000^b^	.755^a^	.570	.000^b^
F=17.446			F = 108.691			F = 125.205			F = 96.592		

a Dependent Variable: Employees’ Satisfaction.

b Predictors: (Constant), Employees’ development: Training, Health and Safety, Employees’ Inclusiveness, Policies against Discrimination, Work-Life Balance.

**Table 5:** Model summary. Source: Researcher׳s Field Survey, 2017.

**Model Summary**			
Multiple *R*	*R* Square	Adjusted *R* Square	Apparent Prediction Error
.755	.570	.564	.430

^a^ Predictors: (Constant), *Employees’ development: Training, Health and Safety, Employees’ Inclusiveness, Policies against Discrimination, Work-Life Balance*.

^b^ Dependent variable: Employees’ Satisfaction.

The above Table 6 shows the statistical significance of the two variables of employees development and employees satisfaction using the categorical regression.

**Table 6:** Model summary (ANOVA^a^). Source: Researcher׳s Field Survey, 2017.

**ANOVA**					
	Sum of Squares	df	Mean Square	*F*	Sig.
Regression	191.526	10	19.153	43.085	.000
Residual	144.474	325	.445		
Total	336.000	335			

^a^ Dependent Variable: Employees’ Satisfaction.

b Predictors: (Constant), Employees’ Development.

Table 7 shows the combined influence of the independent variables (training and development, health and safety, employees’ inclusiveness, policies against discrimination, work-life balance) on employees’ satisfaction (the dependent variable) of the firms. The result in Table 7 further establishes that the composite influence of the firm׳s employees’ development did not occur by chance as it gives the *F*-ratio value of 43.085, which signifies the strength of the four independent variables (under employees’ development) as potent predictors of employee satisfaction of the firms.

**Table 7:** Model summary (coefficients)^a^. Source: Researcher׳s Field Survey, 2017.

	Standardised Coefficients		df	F	Sig.
	Beta	Bootstrap (1000) Estimate of Std. Error			
The firm is committed to employees development through trainings and workshops	.273	.073	1	14.194	.000
The firm has sufficient arrangements for the health and safety of its employees	.162	.090	1	3.272	.001
The company encourages a good work-life balance scheme for its employees (e.g. flexible working hours)	.135	.078	4	2.951	.020
Employees are consulted on important issues	.245	.147	1	2.789	.006
The firm has a policy that avoids discrimination against employees	.463	.086	3	28.739	.000

*Dependent Variable: Employees’ Satisfaction*.

The result in Table 7 shows the staff opinion on employees’ development in improving employees’ satisfaction of oil and gas firms and it reveals that policies against discrimination is a major predictor of employees’ satisfaction which has the highest beta value of (beta = .463, *p* < .005, Sig. .000) than other variables: commitment to employees development through trainings and workshops scaled (beta = .273, *p*< .005, Sig. .000), sufficient arrangements for the health and safety of its employees scaled (beta = .162, *p*< .005, Sig. .004), and encouragement of a good work-life balance scheme for its employees (e.g. flexible working hours) scaled (beta = .135, *p*< .005, Sig. .004). This means that policies against discrimination makes the strongest unique contribution in influencing employee satisfaction. While Employees inclusiveness in important issues is not statistical significant.

## 2 Experimental design, materials and methods

The data presented a quantitative research based on a descriptive research design to assess the effect of employees’ development on employees’ satisfaction within the confines of corporate social responsibility. Survey method was considered appropriate for data gathering.

The population of the study consists of the stakeholders of four (4) top oil and gas firms listed on the Nigerian stock exchange. The choice of these firms is in support of previous studies [1], [2], [3], [4] where it was statistically proffered that the study of corporate social responsibility is best situated in firms with top financial performance, as indicated by high stock price, which invariably means that the firm can carry out its economic obligations, and as such has resources to deal with social problems [5]. In total, there are one thousand, seven hundred and forty eight (1748) employees in all four firms. 350 employees were judiciously selected to partake in this research [6]. Data were collected from these organizations using an adapted researcher made questionnaire. A proportional analysis was conducted to determine the number of copies of the questionnaire to be distributed to the individual firms. The questionnaire is in two sections A and B. Section A contains background questions, section B consists of questions that are specific to the data provided, that is employees’ development and employees’ satisfaction.

The data was coded and keyed into the statistical package for social sciences(SPSS) version 22. Data was described using inferential statistical tests involving multiple regression analysis (Table 8).

**Table 8:** Total number of employees. Source: Human Resource Archives of Selected Oil and Gas firms, 2017.

	**Firm 1**	**Firm 2**	**Firm 3**	**Firm 4**	**Total**
Number of Employees	401	477	485	385	1748

The researchers ascertained that respondents were well informed about the background and the purpose of the research. Every respondent was entitled to the opportunity to stay anonymous and their responses treated with utmost confidentiality. Permission was obtained from the appropriate authorities in the firms where copies of the research instruments were distributed.
