# Supplementary material for: Survey data on employees’ development and employees’ satisfaction in oil and gas firms in Nigeria
Source: Data Brief. 2018 Jun 28;19:1816–21. doi: 10.1016/j.dib.2018.06.066 (PMC6141964; doi:10.1016/j.dib.2018.06.066)
Supplement: Supplementary file 1 — Supplementary material [file mmc1.docx]

**COVER LETTER**

This manuscript describes original work and is not under consideration by any other journal. All authors approved the manuscript and this submission for your consideration for publication in Data in Brief. Please find the enclosed manuscript entitled **Survey Data on Employees’ Development and Employees’ Satisfaction in Oil and Gas Firms in Nigeria**

Bolanle D. Motilewa

[Bolanle.motilewa@covenantuniversity.edu.ng](mailto:Bolanle.motilewa@covenantuniversity.edu.ng)

Oluwatosin C. Bisi-Adeniyi

oluwa[tosin.bisi-adeniyi@covenantuniversity.edu.ng](mailto:tosin.bisi-adeniyi@covenantuniversity.edu.ng)

Oluwaseyi A. Fambegbe

[oluwaseyi.fambegbe@covenantuniversity.edu.ng](mailto:Bolanle.motilewa@covenantuniversity.edu.ng)

Adeola I. Oyeyemi

[Adeola.oyeyemi@covenantuniversity.edu.ng](mailto:Adeola.oyeyemi@covenantuniversity.edu.ng)

Rowland E.K. Worlu

[rowland.worlu@covenantuniversity.edu.ng](mailto:rowland.worlu@covenantuniversity.edu.ng)

Chinonye L. Moses

[chinonye.moses@covenantuniversity.edu.ng](mailto:chinonye.moses@covenantuniversity.edu.ng)

**CA: Oluwaseyi A. Fambegbe**

[**oluwaseyi.fambegbe@covenantuniversity.edu.ng**](mailto:oluwaseyi.fambegbe@covenantuniversity.edu.ng)

**COVENANT UINVERSITY**
