# Supplementary material for: Survey data on employees’ development and employees’ satisfaction in oil and gas firms in Nigeria
Source: Data Brief. 2018 Jun 28;19:1816–21. doi: 10.1016/j.dib.2018.06.066 (PMC6141964; doi:10.1016/j.dib.2018.06.066)
Supplement: Supplementary file 2 — Supplementary material [file mmc2.docx]

# RESEARCH QUESTIONNAIRE ON EMPLOYEES’ DEVELOPMENT AND EMPLOYEES’ SATISFACTION

Dear respondent,

This questionnaire aims to get data on the above topic. I kindly ask for your voluntary co-operation in filling out this questionnaire.

Please note that the exercise is strictly for academic purpose and the information provided will be kept in strict confidence. Response to this questionnaire is absolutely voluntary and kindly note that you can decide at any point in time not to take part in this survey.

Thank you for your anticipated support and co-operation

**(Researcher)**

**SECTION A: DEMOGRAPHIC DATA (Please tick whichever is applicable)**

1. Gender: Male Female
2. Age:
3. Under 25 years b. 25 – 35 years

c. 36 – 45 years d. 46 years and above

1. Length of service in the Oil and Gas Sector
2. Less than 5 years b. 5 – 10 years
3. 11 – 15 years d. 16 years and above
4. Position in the organisation
5. Director b. Senior Manager

c. Supervisor d. Analyst

d. Others, please specify ………………….

1. Please select the category you feel most appropriately describes your unit in the organisation
   1. Engineering b. Administrative

c. Finance and Accounts d. Analyst

Others, please specify ………………….

1. Educational qualification
2. OND/NCE b. HND/BSc.

c. MSc/MBA/M.Ed. d. Others

1. Number of years working in the company:
2. Less than 5 years b. 5 – 10 years
3. 11 – 15 years d. 16 years and above

**Employees’ Development and Employees’ Satisfaction**

| **S/No** | **Item** | **SA** | **A** | **U** | **D** | **SD** |
| --- | --- | --- | --- | --- | --- | --- |
| 1 | There is a commitment to employees development through trainings and workshops in our firm |  |  |  |  |  |
| 2 | Sufficient arrangements for the health and safety of employees is made |  |  |  |  |  |
| 3 | The company encourages a good work-life balance scheme for its employees (e.g. flexible working hours) |  |  |  |  |  |
| 4 | Employees are consulted on important issues |  |  |  |  |  |
| 5 | The firm has a policy that avoids discrimination against employees |  |  |  |  |  |
| 6 | The firm provides incentives and recognises employees’ performance |  |  |  |  |  |
| 7 | The firm is able to attract and retain quality employees |  |  |  |  |  |
| 8 | Staff turnover is minimised in the firm |  |  |  |  |  |
| 9 | Employees are very satisfied with the firm’s work-life balance schemes |  |  |  |  |  |
| 10 | The firm’s policies on employee wellbeing creates a peaceful work environment |  |  |  |  |  |
| 11 | The welfare package in my firm is fair and encouraging |  |  |  |  |  |
